# Influence and significance of bilateral upper-extremity training on recovery of upper-extremity motor function for hemiplegic patients with mild-moderate cerebral apoplexy: a randomised controlled study

**DOI:** 10.4314/ahs.v22i3.40

**Published:** 2022-09

**Authors:** Hongmei Li, Yuanyuan Han, Feng Sheng, Fanliang Kong, Jing Dong

**Affiliations:** Department of Rehabilitation Medicine, Affiliated Hospital of Jilin Medical College, Jilin, China

**Keywords:** Mild-Moderate Cerebral Apoplexy, Bilateral Upper-Extremity Training, Upper-Extremity Motor Function

## Abstract

**Background:**

The recovery of coordination ability of both hands is conductive to improving the activity of daily living for hemiplegic patients.

**Objective:**

To explore the influence and significance of bilateral upper-extremity training on recovery of upper-extremity motor function for hemiplegic patients with mild-moderate cerebral apoplexy.

**Methods:**

Patients were divided into control group and experimental group. The patients in the control group only exercised the upper limbs on the affected side, while the patients in the experimental group exercised the upper limbs on both sides. The Fugl Mayer Assessment Upper Extremity Scale (FMA-UE), Upper Extermities Functional Test (UEFT), modified Barthel index (MBI) and Brunnstrom scores were evaluated in the two groups before and after treatment.

**Results:**

After four weeks, six weeks and eight weeks of treatment, scores of FMA-UE, UEFT, MBI and Brunnstrom for patients increased with the extension of training time, and FMA-UE, UEFT, MBI and Brunnstrom scores for patients of the two groups after four weeks six weeks and eight weeks of treatment showed a significant difference (P<0.05).

**Conclusion:**

The improvement of upper-extremity motor function can be facilitated via relatively conventional training of bilateral upper-extremity training adopted by hemiplegic patients with mild-moderate cerebral apoplexy.

## Introduction

Cerebral apoplexy, characterized by high case fatality rate and high disability rate, is a disease in which oxygen-deficient brain cells are damaged or killed due to the cutoff of blood supply for partial brain.^1^–^3^ After stroke, it will lead to ischemia-hypoxic necrosis and apoptosis of brain tissue around the lesion, strong acid changes in intracellular protein components, oxygen free radical damage and other adverse factors.^4^ Cerebral apoplexy hemiplegia belongs to upper motor neuron damage, i.e. central paralysis. The upper motor neuron is mainly to provide and deliver voluntary movements to the lower motor neuron whose activities can be controlled and dominated by the former, therefore, after cerebral apoplexy, the voluntary movements on the hemiplegic side reduce or disappear.^5^ According to statistics, more than 85% of patients with cerebral apoplexy have upper-extremity dysfunction of different degrees after the onset of disease.^6^ Due to that the motor pattern of upper extremities is elaborate and complex, large joints such as shoulder, elbow and wrist have a higher degree of freedom, the upper-extremity function of patients with cerebral apoplexy usually has a poor recovery and a longer time for recovery, and the adoption of various rehabilitation technologies to recover the upper-extremity function has become one of the major concerns for cerebral apoplexy.^7^,^8^ The recovery of limb motor function in hemiplegia patients is affected by factors such as the repair of their own nerve cells, personal behavior, the external environment and their own experience. Professional rehabilitation therapy can effectively improve the patients ‘ daily activities, reduce the disability rate of the patients, restore the patients’ various functions to the greatest extent, allow the patients to return to the family and society as soon as possible, and reduce the consumption of social resources. 9,10

With the vigorous development of rehabilitation medicine in recent years, the continuous development and innovation of rehabilitation treatment technology, people's understanding of rehabilitation treatment is also constantly improving. The effect of rehabilitation therapy has been gradually recognized by more and more patients and their families. Currently clinically applied rehabilitation technologies, such as neurodevelopmental therapy, motor relearning, compulsory exercise therapy and electronic biofeedback therapy, emphasize the simple movement of the upper limb on the affected side, while ignoring the training of the non-hemiplegic side of the limb, which has certain limitations on the recovery of upper limb function for patients. Most activities in daily life are completed via the coordination between both hands, and the recovery of coordination ability of both hands is conductive to improving the activity of daily living for hemiplegic patients. Moreover, the left and right hemispheres of the brain are connected by the corpus callosum, and they influence and restrain each other. After brain injury, they can still undergo functional reorganization and have high plasticity. This provides the theoretical basis and feasibility of bilateral limb training for this study. In recent years, there are many studies on bilateral limb training for stroke patients, which show that compared with unilateral training, bilateral limb training can also promote the recovery of patients' motor function,^11^ so bilateral limb training has gradually become a research hotspot of post-stroke training methods. This study included the hemiplegic patients with mild-moderate cerebral apoplexy admitted to the Affiliated Hospital of Jilin Medical College, both unilateral and bilateral training methods were used to evaluate the recovery of upper limb function in patients with mild to moderate stroke hemiplegia, and to explore the influence and significance of bilateral upper-extremity training on recovery of upper-extremity function for hemiplegic patients with mild-moderate cerebral apoplexy.

## Data and Methods

### General Data

A total of 160 patients with cerebral apoplexy admitted to the Affiliated Hospital of Jilin Medical College from January 2018 to January 2020 were selected, all of whom conform to the diagnostic standards of cerebral apoplexy. Patients with mild-moderate cerebral apoplexy were divided into control group (n=80) and experimental group (n=80) according to random number table. There's no significant difference between the general data of both groups (P>0.05) via comparison.

#### Inclusion standards

1. Patients with cerebral apoplexy who conform to the diagnostic standards formulated in Essentials for the Diagnoses of Various Cerebrovascular Diseases that is revised in the 4^th^ National Conference on Cerebrovascular Disease of Chinese Medical Association in 2019. Sudden onset of the patient, the common symptoms and signs of cranial nerve injury include hemiplegia, speech and swallowing disturbance, sensory disturbance, hemi-neglect, coordination disturbance, and the diagnosis is confirmed by CT or MRI examination; 2. With the onset for the first time and the course of disease for more than one month; 3. Without obvious consciousness or cognitive disorder; 4. Having signed the informed consent form.

#### Exclusion standards

1. With serious cognitive, movement and affective disorders; 2. With unilateral neglect or visual impairment; 3. Patients with respiratory failure; 4. Patients with congestive heart failure; 5. Patients with active liver disease and hepatic renal insufficiency; patients with malignant tumor; 6. With other rheumatic diseases, rheumatoid diseases or fractures.

### Methods

Drug therapies and symptomatic treatments, such as blood pressure lowering, anticoagulation, blood glucose control and neurotrophy, were administered to both groups. The patients in the control group only exercised the upper limbs on the affected side, while the patients in the experimental group exercised the upper limbs on both sides.

### Upper limb training methods

The therapist trained the patients for one course of treatment, i.e. eight weeks in total, and 15 minutes of separated finger induction training was carried out every day. A patient sat on a back rest chair with a moderate height, avoiding the flexion of wrist joints and maintaining moderate dorsiflexion of wrist joints. The therapist firstly carried out a set of conventional passive activities for the hemiplegic upper extremity and hand, generally including flexion/extension, rotation inward/outward, and abduction/abduction of shoulder joint, flexion/extension of elbow joint, lifting/protraction of straight arm, movement and dorsiflexion of wrist joints and knuckles. The patient put the hemiplegic upper extremity in front of the treatment table, and the flexion of elbow joint was about 30°. If the muscular tension of flexor increased obviously when the upper extremity of patient acted forcibly, with the assistance of the therapist, the upper extremity was straightened, the forearm was rotated forward, and the palm was placed outward in front and outside of the treatment table. The wrist joint was padded with a sandbag to avoid affecting the flexion and extension movements of wrist. The five fingers flexed naturally and slightly and were placed on the treatment table. The training on upper extremity started from the rest position, i.e. the state in which the forearm rotated forward, the wrist flexed, each metacarpophalangeal joint extended, and each interphalangeal joint flexed for at least 10°, so as to carry out the guidance on the action of separated fingers.

### Rating Methods

The scores of Fugl Mayer Assessment Upper Extremity Scale (FMA-UE) 12, Upper Extermities Functional Test (UEFT) 13, Modified Barthel Index (MBI) 14 and Brunnstrom^15^ of both groups before and after treatment were rated. FMA-UE includes a total of 33 items related to proximal and distal limb movements of the upper limbs, and each item is scored on a scale of 0, 1, and 2. A score of 0 is completely unable to perform the required action, a score of 2 is able to perform the required action, and a score of 1 is somewhere in between. The maximum score of FMA-UE is 66, and the higher score indicates the better upper-extremity function. UEFT is divided into four levels: 0–1 points: all activities cannot be completed; 1–2 points: only part of the activity can be completed; 2–3 points: able to complete the activity, but with slow or clumsy movements; 3–4 points: the activity can be completed normally. The full score of UEFT is 99, and the higher score indicates the better function of upper extremities and both hands. The MBI combines evaluation scores from 10 activities on a scale of 0–100. The higher score of MBI indicates the stronger activity of daily living. Brunnstrom scores utilized the available motor pattern to induce the motor response at any time in the recovery process after cerebral injury, so as to stimulate the patient's desire to participate in treatment actively. The rating results before treatment and after two weeks, three weeks, four weeks, six weeks and eight weeks of treatment were observed separately.

### Statistical Analysis

SPAA22.0 statistical software is adopted for analytical processing. The measurement data was expressed as (x±s). Independent-samples t test is used for inter-group comparison, and paired-samples t test is used for intra-group comparison. Enumeration data adopt χ2 test. The significance level takes α= 0.05. P<0.05 represents that the data between both groups have significant differences.

## Results

### FMA-UE Scores

The baseline data of the two groups of patients are shown in Table 1. There is no significant difference in the clinical characteristics between the groups. Before treatment, FMA-UE scores between both groups showed no significant difference (P =0.772). After two weeks of treatment, FMA-UE scores of the experimental group increased compared with that before treatment, but showed no significant difference (P > 0.05); after four weeks of treatment, FMA-UE scores of both groups increased compared with those before treatment (P < 0.05), and the scores of the experimental group were significantly higher than those of the control group (P=0.001); after six weeks of treatment, FMA-UE scores of both groups increased significantly compared with those after four weeks of treatment (P < 0.05), and the scores of the experimental group were significantly higher than those of the control group (P =0.029); after eight weeks of treatment, FMA-UE scores of both groups increased significantly compared with those after six weeks of treatment (P < 0.05), but the scores between experimental group and control group showed no significant difference (P =0.173). FMA-UE scores presented a gradual increase trend with the prolongation of treatment time. Refer to Table 2.

**Table 1:** Comparison of General Data of Both Groups

Variable	Control Group (n=80)	Experimental Group (n=80)	P
Age	59.03±9.014	58.98±8.256	0.894
Gender			
Male	41	43	
Female	39	37	0.614
Course of Disease	45.02±6.05	48.34±7.25	0.213
Hemiplegic Side			
Left Side	39	43	
Right Side	41	37	0.603

**Table 2:** Comparison of FMA-UE Scores Between Experimental Group and Control Group

Group	n	Before Treatment	After Two Weeks of Treatment	After Four Weeks of Treatment	After Six Weeks of Treatment	After Eight Weeks of Treatment
Control Group	80	15.16±3.51	17.45±3.89	21.63±4.63*^a^	26.59±5.86*^a^^b^	29.26±4.15*^a^^b^^c^
Experimental Group	80	15.31±3.01	17.56±3.56	24.05±4.53*^a^	28.51±5.16*^a^^b^	30.19±4.43*^a^^b^^c^
t		0.192	0.915	3.342	2.199	1.370
P		0.772	0.342	0.001	0.029	0.173

FMA-UE, Fugl Mayer Assessment Upper Extremity Scale.

* P<0.05 VS Before treatment

a P<0.05 VS After two weeks of treatment

b P<0.05 VS After four weeks of treatment

c P<0.05 VS After six weeks of treatment.

### UEFT Scores

Before treatment, there was no significant difference in UEFT scores between the two groups (P > 0.05). After 2 weeks of treatment, the UEFT scores of the two groups were improved compared with those before treatment, but there was no significant difference compared with before treatment (P > 0.05), and there was no significant difference between the groups (P > 0.05). After four weeks of treatment, UEFT scores of both groups had improvement compared with those before treatment (P < 0.05), and the scores of the experimental group were higher than those of the control group (P < 0.001). After six weeks and eight weeks of treatment, UEFT scores of both groups had further improvement compared with those after four weeks of treatment (P < 0.05), and the scores of the experimental group were higher than those of the control group (P < 0.001). Refer to Table 3.

**Table 3:** Comparison of UEFT Scores Between Experimental Group and Control Group

Group	n	Before Treatment	After Two Weeks of Treatment	After Four Weeks of Treatment	After Six Weeks of Treatment	After Eight Weeks of Treatment
Control Group	80	31.76±5.39	33.02±5.67	40.26±5.31*^a^	47.39±5.23*^a^^b^	52.36±4.93*^a^^b^^c^
Experimental Group	80	32.95±5.54	35.54±5.21	44.85±5.08*^a^	51.56±5.33*^a^^b^	55.62±5.03*^a^^b^^c^
t		0.943	1.265	5.587	5.043	4.140
P		0.351	0.169	<0.001	<0.001	<0.001

UEFT, Upper Extermities Functional Test.

* P<0.05 VS Before treatment

a P<0.05 VS After two weeks of treatment

b P<0.05 VS After four weeks of treatment

c P<0.05 VS After six weeks of treatment.

### MBI Scores

Before treatment, there was no significant difference in MBI scores between the two groups (P > 0.05). After 2 weeks of treatment, the MBI scores of the two groups were improved compared with those before treatment, but there was no significant difference compared with before treatment (P > 0.05), and there was no significant difference between the groups (P > 0.05). After four weeks of treatment, MBI scores of both groups had improvement compared with those before treatment (P < 0.05), and the scores of the experimental group were higher than those of the control group (P =0.002). After six weeks and eight weeks of treatment, MBI scores of both groups had further improvement compared with those after four weeks of treatment (P < 0.05), and the scores of the experimental group were higher than those of the control group (P < 0.01). Refer to Table 4.

**Table 4:** Comparison of MBI Scores Between Experimental Group and Control Group

Group	n	Before Treatment	After Two Weeks of Treatment	After Four Weeks of Treatment	After Six Weeks of Treatment	After Eight Weeks of Treatment
Control Group	80	38.19±5.79	40.26±5.92	48.65±6.73*^a^	53.48±6.99*^a^^b^	57.09±7.40*^a^^b^^c^
Experimental Group	80	37.95±6.59	41.56±7.12	52.49±8.84*^a^	56.98±8.86*^a^^b^	61.29±8.91*^a^^b^^c^
t		0.271	1.145	3.091	2.788	3.243
P		0.782	0.162	0.002	0.006	0.001

MBI, Modified Barthel Index.

* P<0.05 VS Before treatment

a P<0.05 VS After two weeks of treatment

b P<0.05 VS After four weeks of treatment

c P<0.05 VS After six weeks of treatment.

### Brunnstrom Scores

Before treatment, Brunnstrom scores of both groups showed no significant difference (P =0.425). After two weeks of treatment, Brunnstrom scores of both groups increased significantly compared with those before treatment (P<0.05), but the comparison between control group and experimental group showed no significant difference (P=0.172). After four weeks of treatment, Brunnstrom scores of both groups had significant improvement compared with those after two weeks of treatment (P < 0.05), and the scores of the experimental group were higher than those of the control group (P= 0.05). After six weeks and eight weeks of treatment, Brunnstrom scores of both groups had further improvement (P < 0.05), and the scores of the experimental group were higher than those of the control group (P < 0.05). Refer to Table 5.

**Table 5:** Comparison of Brunnstrom Scores Between Experimental Group and Control Group

Group	n	Before Treatment	After Two Weeks of Treatment	After Four Weeks of Treatment	After Six Weeks of Treatment	After Eight Weeks of Treatment
Control Group	80	35.05±10.65	43.80±10.64*	58.36±10.32*^a^	65.15±10.02*^a^^b^	71.55±10.56*^a^^b^^c^
Experimental Group	80	35.11±10.64	44.85±10.63*	59.99±10.41*^a^	67.13±10.52*^a^^b^	74.55±10.45*^a^^b^^c^
T Value		0.571	1.153	2.235	2.353	2.548
P Value		0.425	0.172	0.035	0.025	0.019

* P<0.05 VS Before treatment

a P<0.05 VS After two weeks of treatment

b P<0.05 VS After four weeks of treatment

c P<0.05 VS After six weeks of treatment.

## Discussion

Hemiplegia is the most common sequela of stroke. According to statistics, 55%–75% of hemiplegic patients will leave upper limb dysfunction. Compared with lower limbs, the rehabilitation of upper limbs is often more time-consuming and less effective.^16^ Stroke hemiplegia seriously reduces the quality of life of patients, and also increases the burden on the patient's family and society. Therefore, how to improve the limb dysfunction of stroke hemiplegia patients is a key clinical concern. Due to the unstable condition of stroke patients in the early stage, they can only rest in bed and cannot move. After a week of bed rest, the muscles of the limbs will shrink by about 20%, resulting in decreased muscle strength. Rehabilitation treatment should comprehensively improve the patient's function. The early decline of the muscle strength of the contralateral limb will also affect the recovery of the patient's overall function, so it is necessary to carry out bilateral limb training for stroke patients.

Neuroplasticity and functional reorganization are theoretical bases for the rehabilitation of hand function. At present, according to the above two theories and the difference of site of action for treatment, the upper-extremity rehabilitation treatment is divided into peripheral intervention and central intervention.^17^ Primarily based on the plasticity reactivation of brain, the peripheral intervention realizes rehabilitation via the extremity movement activation of the affected side, which mainly includes constraint-induced movement therapy and euromuscular facilitation technique. 18 Central intervention directly positions and stimulates cerebral cortex, and adjusts the functional equilibrium of brain, which includes motor imagery therapy and mirror therapy. 18 This paper mainly studies the influence and significance of bilateral upper-extremity training on the recovery of upper-extremity motor function for hemiplegic patients with mild-moderate cerebral apoplexy in peripheral intervention therapy. The study of Lee M et al.^19^ found that the combination of bilateral upper-extremity training and conventional rehabilitation treatment was more effective than the pure rehabilitation treatment in improving the upper-extremity function and activities of daily living performance of patients. Renner CIE et al. 20 studied bilateral arm training vs unilateral arm training in severe stroke patients and found that Fugl-Meyer Assessment (FMA) scores were significantly higher in patients with bilateral arm training than in patients with unilateral arm training. The results of this study showed that the use of bilateral upper limb training significantly improved the upper limb motor function in patients with mild to moderate stroke hemiplegia compared with unilateral upper limb training. Compared with before treatment, after 4 weeks of treatment, patients' FMA-UE, UEFT, MBI and Brunnstrom scores were significantly improved, and the above scores of patients in the bilateral upper extremity training group were significantly due to the patients in the unilateral training group.

Combining with the domestic and foreign literatures in recent years, this paper considers that the possible mechanism of bilateral rehabilitation training is as follows.

1. Disinhibition of bilateral cerebral hemispheres: Gandolfi et al.^21^ thought that one of the underlying mechanisms for the positive influence of bilateral training was that the inhibitory circuit in the cortex between cerebral hemispheres changed, thus facilitating the influence of cerebral hemispheres on motor function of extremities. Stinear et al.^22^ carried out the repetitive training on dorsiflexion movement of wrist joints for patients with cerebral apoplexy via Bi-Manu-Tract (BMT). The results showed that such training had direct influence on the activation of relative neurons in motor cortical area of wrist extensor and considered that the treatment effect could be magnified by activating and rebalancing the inhibition between the two hemispheres. 2. Body control: Compared with the upper-extremity task training of the affected side, the bilateral arm training causes more trunk muscle contractions, thus better controlling the proximal upper extremity,^23^ meanwhile, the increase of movement intensity can facilitate the expression of brain-derived neurotrophic factors and brain function remodeling to some extent.^24^ 3. Feedback adaption: Through the repetitive and dense bilateral movement training, the normal proprioception is taken as the feedback to complete the input of action information, activate more neural pathways, stimulate the damaged area to the maximum extent, and facilitate the remodeling of structure and function of damaged neurons.^25^ The combination with neurophysiological adaptation can facilitate the plasticity of brain function and help patients to recover the movement ability of upper extremities. The results of this study show that the adoption of bilateral upper-extremity training based on conventional training can significantly improve the upper-extremity motor function of hemiplegic patients with mild-moderate cerebral apoplexy. Some studies consider that the neuroplasticity degree is closely related to factors such as movement training frequency, intensity, time, pattern, content, peripheral stimulation and sensory feedback.^26^ After adopting the bilateral upper-extremity training, the patients have upper-extremity motor function, activities of daily living, movement control ability and degree of activation in the damaged cortical area that are superior to the conventional intensive movement training on upper extremity of the affected side.

## Conclusion

Upper limb rehabilitation training can improve the recovery of upper limbs in stroke patients. Compared with unilateral upper limb training, bilateral upper limb training can better promote the recovery of upper limb motor function in hemiplegic patients.
